# Conditional survival estimate in patients with Barcelona Clinic Liver Cancer stage B/C hepatocellular carcinoma treated with hepatic arterial infusion chemotherapy with/without concurrent radiotherapy

**DOI:** 10.18632/oncotarget.20321

**Published:** 2017-08-18

**Authors:** In Rae Cho, Hye Won Lee, Ki Jun Song, Beom Kyung Kim, Seung Up Kim, Do Young Kim, Sang Hoon Ahn, Jinsil Seong, Kwang-Hyub Han, Jun Yong Park

**Affiliations:** ^1^ Department of Internal Medicine, Yonsei University College of Medicine, Seoul, Korea; ^2^ Yonsei Liver Center, Severance Hospital, Seoul, Korea; ^3^ Institute of Gastroenterology, Yonsei University College of Medicine, Seoul, Korea; ^4^ Department of Biostatistics, Yonsei University College of Medicine, Seoul, Korea; ^5^ Department of Radiation Oncology, Yonsei University College of Medicine, Seoul, Korea

**Keywords:** advanced hepatocellular carcinoma, Barcelona Clinic Liver Cancer stage B/C, conditional survival, hepatic arterial infusion chemotherapy, concurrent chemoradiotherapy

## Abstract

Conditional survival (CS) provides a prognosis of patients who have already survived several years after treatment. We investigated CS in Barcelona Clinic Liver Cancer (BCLC) stage B/C hepatocellular carcinoma (HCC) patients treated with hepatic arterial infusion chemotherapy (HAIC) with or without concurrent radiotherapy (CRT). A total of 181 patients diagnosed with HCC who were treated with HAIC with or without CRT between 2011 and 2015 were retrospectively reviewed. Overall survival (OS) and CS were calculated and a subgroup analysis was performed. The 1- and 5-year survival rates of all patients were 57.0% and 24.3%. OS was significantly higher in patients with BCLC stage B than BCLC stage C patients. Patients who achieved disease control after treatment also showed longer OS than who did not respond to treatment. Provided that the patient had already survived for 0, 1, 2, and 3 years, the CS estimates of surviving an additional 2 years were 35.6%, 55.1%, 82.0%, and 77.4%, respectively. A subgroup analysis was performed to compare BCLC stage B and C patients and revealed that CS has a tendency to increase and the difference in CS between two groups decreased over time. CS reflects the change of prognosis over time and may provide a more accurate prognosis and hopeful message to patients who have already survived with treatment.

## INTRODUCTION

Hepatocellular carcinoma (HCC) is the sixth most common cancer in the world and the second most common cause of cancer-related mortality [1]. The incidence of HCC is high in countries where hepatitis B virus (HBV) infection is endemic, such as southeast Asia and sub-Saharan Africa [2]. In Korea, the incidence of HCC is declining, but HCC is still one of the main causes of cancer-related deaths. Although early-stage HCC patients have a good prognosis, with a 60–70% 5-year survival rate, only 30–60% of HCC patients are diagnosed at an early stage [3, 4]. Most HCC patients diagnosed in advanced stage have extensive tumor burden, vascular invasion, extrahepatic spread, and/or decompensated liver function. Patients with advanced HCC have a poor prognosis, with 5-year survival rates of 10.7% in locally advanced stage patients and 3.1% in patients with distant metastasis [5].

Cancer staging and risk stratification are required to determine the optimal treatment strategy and to improve treatment outcomes in HCC patients. The Barcelona Clinic Liver Cancer (BCLC) staging system, which incorporates the patient’s performance status, tumor burden, and liver function, is widely accepted and used among several staging systems [6, 7]. Patients with advanced HCC are classified as BCLC stage B or C; these patients are not candidates for curative treatments, such as surgical resection or radiofrequency ablation (RFA). For this reason, transhepatic arterial chemoembolization (TACE) or systemic therapy with sorafenib are recommended for BCLC stage B/C HCC patients [8]. However, there are some discrepancies in treatment strategies because of regional differences in HCC (i.e., the etiology) and other diversities (e.g., diagnosis and staging) secondary to a lack of high-level evidence [4].

Many efforts have been made to treat advanced HCC patients. Hepatic arterial infusion chemotherapy (HAIC) has proven to be both effective and safe [9–14]. HAIC can achieve increased local drug concentrations at the tumor while reducing systemic exposure and side effects. Therefore, HAIC has been considered a useful palliative therapeutic option for advanced HCC patients. Concurrent chemoradiation therapy (CCRT) was recently introduced. In our institution, localized CCRT followed by HAIC has been used to treat advanced HCC without extrahepatic metastasis and has shown promising results [15]. Some patients treated with CCRT (HAIC with CRT) exhibit good treatment responses and conversion from advanced to resectable HCC [16–18].

Advanced HCC patients who exhibit a favorable treatment response with CCRT (or HAIC) and have already survived several years after treatment have a different probability of survival than is estimated at the time of diagnosis. In these patients, prognosis is more accurately described using conditional survival (CS) analysis [19]. CS provides a prognosis of patients who have already survived several years after treatment and is a useful tool to dynamically adjust to individualized survival. A more accurate individual prognosis derived from CS estimates would be important in clinical practice and in research. Conditional survival estimates have been published for patients with various cancers [20–22] and HCC with curative treatments [23, 24]. However, CS data from advanced HCC patients with palliative treatment are limited.

The present study explored how CS probability changes over time according to different prognostic variables, focusing on BCLC stage B/C HCC patients treated with HAIC with or without CRT.

## RESULTS

### Patient characteristics

Table 1 lists the baseline characteristics of all 181 patients. The median age was 55 years (range, 28–82 years) and 151 (83.4%) patients were males. The most common etiology of HCC was HBV infection (*n* = 153, 84.5%). The median radiologic tumor size at diagnosis was 9 cm (range, 2–20 cm), and up to 5 masses were found (median, 1; range, 1–5). The median AFP, albumin, and total bilirubin levels were 1444 ng/mL (range, 0.34–120000 ng/mL), 3.9 g/dL (range, 2.0–4.9 g/dL), and 0.9 mg/dL (range, 0.3–28.3 mg/dL), respectively. In total, 159 (87.8%) patients with preserved liver function were in Child-Pugh class A and 22 (12.2%) patients were in Child-Pugh class B. Sixty-three (34.8%) patients were BCLC stage B and 118 (65.2%) patients were BCLC stage C. Approximately 60.8% of patients had vascular invasion at diagnosis. Although 42 (23.2%) patients were treated with HAIC only, most patients (139, 76.8%) were treated with HAIC and CRT.

**Table 1:** Baseline characteristics of all patients

Variables	
Age, years	55 (28–82)
Sex, Male/Female	151/30
Etiology, HBV/Other	153/28
Radiologic tumor size at diagnosis, cm	9 (2–20)
Tumor number at diagnosis	1 (1–5)
AFP at diagnosis, ng/ml	1444 (0.34–120000)
Prothronbin time-INR	1.06 (0.85–1.67)
Albumin (g/dL)	3.9 (2.0–4.9)
Total bilirubin (mg/dL)	0.9 (0.3–28.3)
Platelet count (10^3^/μL)	185 (37–650)
Child-Pugh, A/B	159/22
BCLC, B/C	63/118
TNM stage, II/III/IV	35/67/79
Positive vascular invasion	110 (60.8%)
Treatment modality	
HAIC	42 (23.2%)
HAIC + CRT	139 (76.8%)

*Variables are expressed as median (range) or *n* (%).

*HBV, Hepatitis B virus; INR, International normalized ratio; BCLC, Barcelona Clinic Liver Cancer; HAIC, Hepatic arterial infusion chemotherapy; CRT, Concurrent radiotherapy.

### Treatment efficacy and patient actuarial overall survival

**Figure 1:**
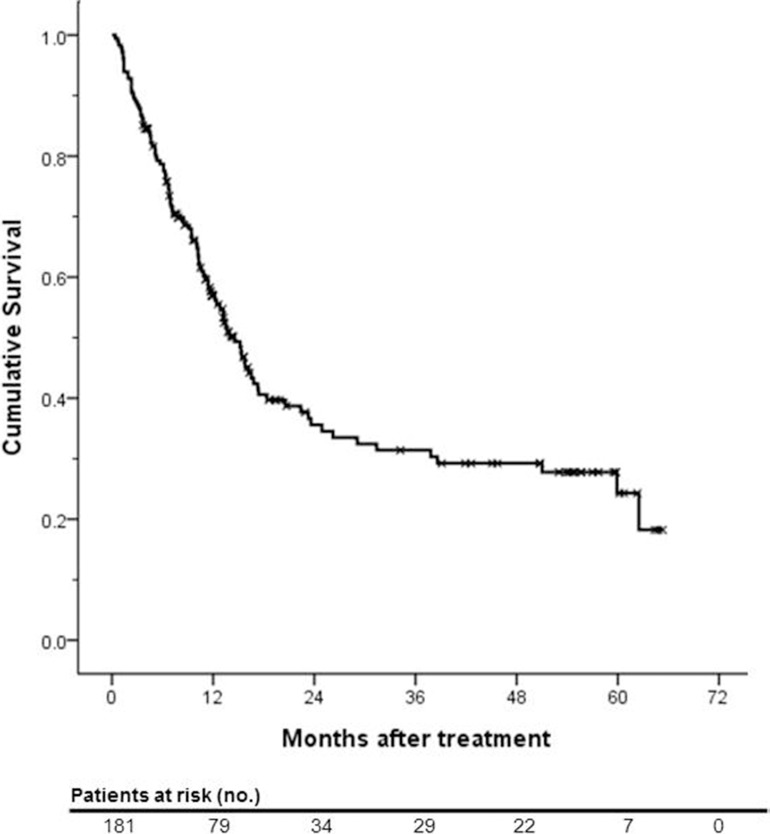
Kaplan-Meier survival curve of actuarial overall survival of the entire study population

**Figure 2:**
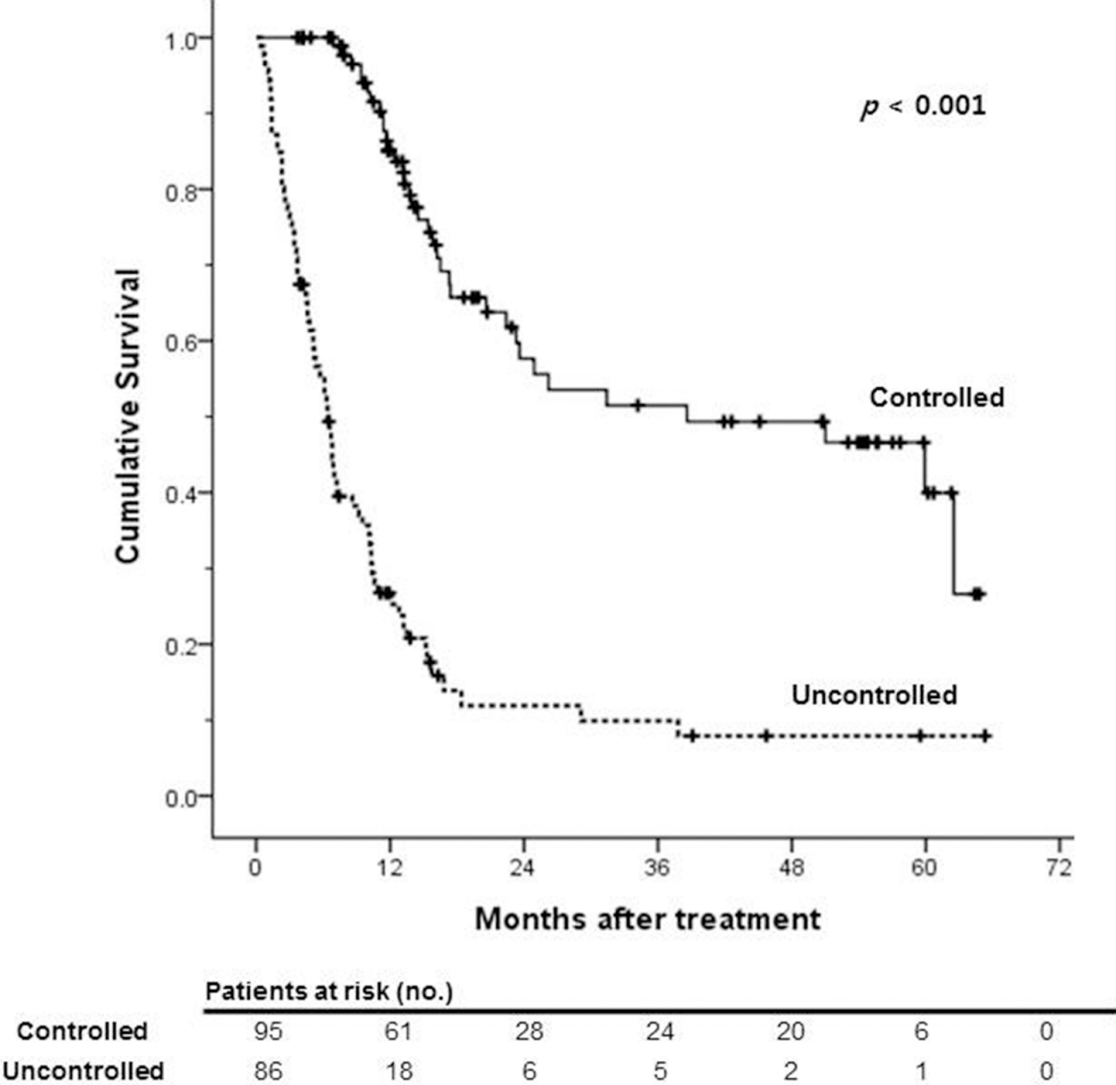
Kaplan-Meier survival curves of overall survival according to the treatment response

The median follow-up period was 11.1 months (range, 0.4–65.3 months). During this period, 107 (59.1%) patients died. the 1-, 3- and 5- year survival rates were 57.0%, 31.4%, and 24.3%, respectively (Figure 1). Treatment response of HAIC with or without CRT is summarized in Table 2. 69 patients (38.1%) showed partial response (PR), 50 patients showed stable disease (SD) and 50 (27.6%) patients showed disease progression (PD). Among all subjects, 38.1% of patients experienced the objective response and disease control rate was 52.5%. The median OS was significantly lower in uncontrolled patients than patients who achieved disease control after HAIC with or without CRT (6.4 months versus 38.6 months, *p* < 0.001; Figure 2).

**Table 2:** Treatment response, objective response rate and disease control rate of all patients

Variables	
Treatment response	
Complete response	0
Partial response	69 (38.1%)
Stable disease	50 (27.6%)
≥ 16 weeks	26 (14.4%)
< 16 weeks	24 (13.2%)
Disease progression	50 (27.6%)
Could not be evaluated	12 (6.6%)
Objective response rate	38.1%
Disease control rate	52.5%

Table 3 lists actuarial survival rates, in relationship with patient characteristics. The median OS was significantly lower in BCLC stage C patients than stage B patients (12.8 versus 24.9 months, *p* = 0.012). Patients in Child-Pugh class A (*p* < 0.001), TNM stage II/III (*p* < 0.001) and those without vascular invasion (*p* = 0.03) had significantly higher survival rates than in patients with Child-Pugh class B, TNM stage IV and vascular invasion. Younger patients and non-B-viral HCC patients tended to have higher survival rates at every time point, but age, gender, HCC etiology, and AFP level at diagnosis did not significantly affect actuarial survival rates.

**Table 3:** Actuarial overall survival rates of patients in relationship to patients’ characteristics

Variables	Patients Survival					
	1 yr	2 yr	3 yr	4 yr	5 yr	*P*
All patients (*n* = 181)	57.0%	35.6%	31.4%	29.2%	24.3%	-
Age						0.239
< 65 (*n* = 137)	58.9%	38.0%	33.9%	32.5%	26.4%	
≥ 65 (*n* = 44)	50.8%	29.1%	24.3%	19.4%	19.4%	
Gender						0.529
Male (*n* = 151)	58.2%	35.7%	33.1%	31.7%	25.6%	
Female (*n* = 30)	51.5%	34.8%	24.9%	19.9%	19.9%	
Etiology						0.057
HBV (*n* = 153)	53.8%	31.5%	27.8%	27.8%	22.0%	
Other (*n* = 28)	74.2%	58.5%	51.2%	43.9%	43.9%	
Child-Pugh						< 0.001
A (*n* = 159)	61.3%	38.3%	33.4%	33.4%	27.8%	
B (*n* = 22)	26.0%	15.6%	15.6%	5.2%	5.2%	
BCLC						0.012
B (*n* = 63)	65.6%	52.4%	43.1%	43.1%	38.8%	
C (*n* = 118)	52.4%	26.9%	25.4%	22.0%	16.5%	
TNM						< 0.001
II/III (*n* = 102)	67.6%	50.2%	42.4%	40.4%	31.6%	
IV (*n* = 79)	43.0%	17.1%	17.1%	15.0%	15.0%	
Vascular invasion						0.03
No (*n* = 71)	60.9%	51.2%	42.1%	42.1%	37.9%	
Yes (*n* = 110)	54.5%	27.1%	25.5%	22.1%	16.6%	
AFP						0.12
< 400 (*n* = 77)	65.1%	40.9%	36.3%	34.1%	20.8%	
> 400 (*n* = 104)	50.8%	31.5%	27.5%	25.4%	25.4%	
Disease control						< 0.001
Controlled (*n* = 95)	85.1%	57.7%	51.5%	49.3%	39.9%	
Uncontrolled (*n* = 86)	26.8%	11.9%	9.9%	7.9%	2.4%	

### Patient survival according to the Hong Kong Liver Cancer staging system

A new HCC staging system was developed by Yau T. et al. in 2014 [25]. This Hong Kong Liver Cancer (HKLC) staging system subdivides BCLC stage B and C patients and recommends more aggressive treatment than the BCLC algorithm. BCLC stage B group patients were distributed from HKLC stage I to III, and BCLC stage C group patients were distributed from HKLC stage II to V. The median OS of HKLC stage II (IIa and IIb), III (IIIa and IIIb), and IV (IVa and IVb) patients was in 31.2∼33.4, 5.5∼12.5, and 1.9∼4.0 months, respectively [25].

**Figure 3:**
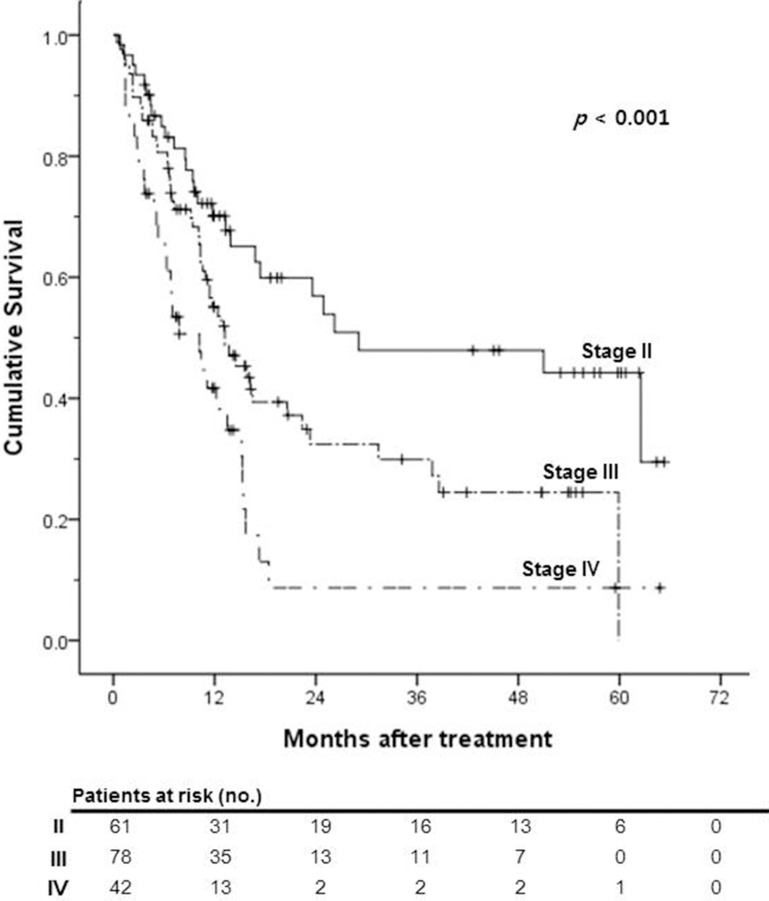
Kaplan-Meier survival curves of overall survival according to the Hong Kong Liver Cancer (HKLC) stage

Survival analysis was performed to evaluate the validity of the HKLC staging system in this study population and to compare the treatment outcomes of our institution (HAIC ± CRT) with the HKLC treatment algorithm. The median OS of HKLC stage II, III and IV patients in our study population was 29.1, 13.2, and 10.2 months, respectively (*p* < 0.001; Figure 3). The difference in OS was statistically significant according to the HKLC stage. Thus, we verified the validity of the HKLC staging system in our study population and the non-inferiority of treatment outcomes of our institution.

### Conditional survival

To predict the effects of BCLC stage and other variables at different time points during the follow-up, the 2-year CS probability after 1, 2 and 3 years was estimated in patients treated with HAIC ± CRT (Table 4). The probability of surviving an additional 2 years, given that the patient had already survived for 1, 2, and 3 years, was 55.1%, 82.0%, and 77.4%, respectively, in the entire study population.

**Table 4:** 2-year conditional survival rates in relationship to patients’ characteristics

Variables	Estimated 2-year conditional survival			
	At diagnosis	1 year after treatment	2 years after treatment	3 years after treatment
All patients (*n* = 181)	35.6%	55.1%	82.0%	77.4%
Age				
< 65 (*n* = 137)	38.0%	57.6%	85.5%	77.9%
≥ 65 (*n* = 44)	29.1%	47.8%	66.7%	79.8%
*d*	0.19	0.20	0.45	−0.05
Gender				
Male (*n* = 151)	35.7%	56.9%	88.8%	77.3%
Female (*n* = 30)	34.8%	48.3%	57.2%	79.9%
*d*	0.02	0.17	0.76	−0.06
Etiology				
HBV (*n* = 153)	31.5%	51.7%	88.3%	79.1%
Other (*n* = 28)	58.5%	69.0%	75.0%	85.7%
*d*	-0.56	–0.36	0.35	−0.17
Child-Pugh				
A (*n* = 159)	38.3%	54.5%	87.2%	83.2%
B (*n* = 22)	15.6%	60.0%	33.3%	33.3%
*d*	0.53	–0.11	1.32	1.17
BCLC				
B (*n* = 63)	52.4%	65.7%	82.3%	90.0%
C (*n* = 118)	26.9%	48.5%	81.8%	65.0%
*d*	0.54	0.35	0.01	0.63
TNM				
II/III (*n* = 102)	50.2%	62.7%	80.5%	74.5%
IV (*n* = 79)	17.1%	39.8%	87.7%	87.7%
*d*	0.75	0.47	−0.20	−0.34
Vascular invasion				
No (*n* = 71)	51.2%	69.1%	82.2%	90.0%
Yes (*n* = 110)	27.1%	46.8%	81.5%	65.1%
*d*	0.51	0.46	0.02	0.63
AFP				
< 400 (*n* = 77)	40.9%	55.8%	83.4%	57.3%
> 400 (*n* = 104)	31.5%	54.1%	80.6%	92.4%
*d*	0.20	0.03	0.07	−0.88
Disease control				
Controlled (*n* = 95)	57.7%	60.5%	85.4%	77.5%
Uncontrolled (*n* = 86)	11.9%	36.9%	66.4%	24.2%
*d*	1.10	0.49	0.46	1.26

**Figure 4:**
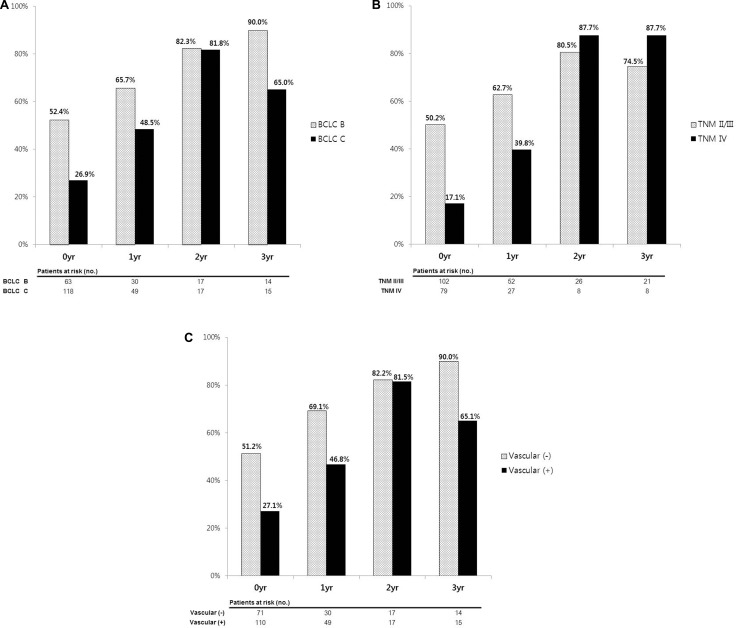
2-year conditional survival rates according to (**A**) BCLC stage, (**B**) TNM stage and (**C**) vascular invasion. The CS difference between two groups was decreased over time.

Both BCLC stage B and C patients tended to have an increased 2-year CS. The 2-year CS of 0, 1, 2, and 3 years in BLCL stage B patients was 52.4%, 65.7%, 82.3%, and 90.0%, respectively, and that in BCLC stage C patients was 26.9%, 48.5%, 81.8%, and 65.0%, respectively (Figure 4A). Standardized differences in the 2-year CS between BCLC B and C groups revealed a tendency to decrease. Standardized differences at 0, 1, 2, and 3 years were 0.54, 0.35, 0.01, and 0.63, respectively, indicating that the BCLC stage of patients at diagnosis does not affect patient survival more than 2 years after treatment. Other factors that reflect tumor aggressiveness, such as TNM stage and the presence of vascular invasion, had a similar tendency to BCLC stage (Figure 4B and 4C). For example, the 2-year CS rates of patients in TNM stage II/III were higher than 20% compared with stage IV patients at 1 year after treatment (62.7% versus 39.8%; *d* = 0.47). However, after 2 years, the CS rate was similar between the two groups and standardized differences were lower than that at 1 year after treatment (*d* = −0.20 and −0.34 at 2 and 3 years after treatment, respectively).

In contrast, treatment response to HAIC ± CRT and baseline liver function did not exhibit a similar change in 2-year CS or standardized differences. Patients who experienced disease control showed better 2-year CS in every time points than patients who had uncontrolled disease (57.7%, 60.5%, 85.4% and 77.5% versus 11.9%, 36.9%, 66.4% and 24.2% at 0, 1, 2, and 3 years, respectively). The standardized differences between two groups did not decrease over time. (*d* = 1.10, 0.49, 0.46 and 1.26 at 0, 1, 2, and 3 years, respectively). Patients in Child-Pugh class A also had a better 2-year CS than more decompensated patients (38.3%, 54.5%, 87.2%, and 83.2% versus 15.6%, 60.0%, 33.3%, and 33.3% at 0, 1, 2, and 3 years, respectively) and the standardized differences did not reveal a tendency to decrease over time (*d* = 0.53, −0.11, 1.32, and 1.17 at 0, 1, 2, and 3 years, respectively).

Although age and HCC etiology did not exhibit a statistically significant difference in actuarial survival rate, younger patients and non-B-viral patients had a tendency to have a better 2-year CS than older and B-viral HCC patients.

## DISCUSSION

We found that tumor characteristics known to affect a patient’s actuarial survival, such as BCLC stage, TNM stage, and vascular invasion, did not affect CS after 2 years of treatment. Patients with more aggressive tumor features who were classified as advanced (higher) stage were expected to have a poor prognosis, and did exhibit a significantly lower actuarial OS rate than early stage patients. However, advanced stage patients who exhibited a favorable treatment response with HAIC ± CRT for the first 2 years had similar CS estimates as patients with less aggressive tumor features.

To date, treatment outcomes and prognosis of cancer patients are typically described as actuarial OS or 5-year survival rate. However, these measures only reflect a patient’s information at the time of diagnosis and represent the survival data of the entire patient population. Conversely, CS provides useful information on the patient’s prognosis that is adjusted for the time the patient already survived. CS provides cancer patients with more individualized prognostic information and determines how their prognosis evolves over time. Merrill et al. demonstrated that CS has a tendency to increase over time in various cancers, and that an increment in CS is notable in advanced stage and more lethal types of cancer (i.e., lung and pancreatic cancer) [26]. This result has been observed for many cancer entities in various studies [20–24, 27–29]. Therefore, CS information can be considered relevant and may provide hope to patients, clinicians, and researchers interested in the probability of surviving additional years.

The prognosis of advanced-stage HCC patients (including locally advanced and unresectable HCC) had been reported as dismal. The median survival of untreated HCC patients with BCLC stage C is 7 months [30] and only 2.7 months in those with portal vein thrombosis (PVT) [31]. Although sorafenib is a recommended treatment option for patients, it is not widely used as first-line treatment because of its marginal survival benefit, relatively high costs, and low tolerability with frequent adverse events [32]. Furthermore, the presence of PVT is a significant limitation of HCC treatment because it is considered a contraindication for transplantation, curative resection, and TACE in treatment guidelines [8, 33]. There is also a problem in establishing treatment strategy for BCLC stage B patients. Although BCLC stage B includes an extremely diverse set of patients, BCLC staging system recommends only TACE as treatment modality. To overcome this problem, studies had been conducted for the appropriate treatment for BCLC stage B patients. Bolondi et al. showed poor prognosis in patients beyond the up-to-7 criteria and suggested the use of sorafenib or clinical trials for patients in substage B2 and B3 [34]. Yamakado et al. revealed that BCLC stage B patients who had ≥ 5 tumors or > 7 cm sized tumor showed markedly reduced treatment response to TACE [35]. In this study population, most patients exhibited advanced HCC at diagnosis. Approximately 65.2% (*n* = 118) of patients were in BCLC stage C and 60.8% (*n* = 110) of patients had vascular (portal vein [PV] and hepatic vein [HV]) tumor invasion. Even in BCLC stage B patients of this study population (*n* = 63) who did not showed any vascular invasion, most of patients had multiple or massive, infiltrative tumors larger than 5 cm that were not suitable for loco-regional therapy.

To treat advanced HCC patients, our institution used a combination therapy of HAIC and CRT on the basis of previous favorable treatment results [15]. Although patients who had a diffuse or multifocal bi-lobal tumor could not be treated with radiotherapy to avoid whole-liver irradiation, which could cause serious adverse effects, patients with PVT (including the main portal vein) were successfully treated with HAIC ± CRT and exhibited tolerable treatment-related side effects. Similar to previous treatment outcomes, HAIC ± CRT yielded notable treatment outcomes in this study. The median survival of BCLC stage C patients in this study group was 12.8 months and that in patients with vascular invasion was 13.2 months. HAIC allows the local delivery of chemotherapeutic agents in high concentrations and maximizes the therapeutic effects of radiation therapy with reduced systemic toxicity [10, 36–38]. Excellent tumor responses, include down-staging of HCC, could be achieved by HAIC ± CRT. Therefore, it could be a useful treatment modality in advanced HCC patients.

We also reviewed the medical records regarding tumor characteristics at diagnosis and treatment outcomes of long-term survivors who survived for 2 or more years. In total, 34 patients survived for 2 or more years. Among them, 32 (94.1%) patients exhibited favorable treatment responses after HAIC ± CRT: 18 patients eventually achieved successful down-staging followed by curative operation (including 4 patients who underwent liver transplantation) and 1 patient achieved a complete response (no residual tumor on imaging) after HAIC with CRT and consecutive additional HAIC. Of the long-term survivors, 50% of patients were BCLC stage C patients and 50% had vascular invasion of the tumor at diagnosis. Considering the poor prognosis of advanced HCC patients, these proportions are meaningful. We previously demonstrated that BCLC stage and vascular invasion of the tumor at diagnosis does not affect patient survival more than 2 years after treatment. Therefore, a good prognosis could be expected for patients who have favorable treatment responses, even for those with advanced HCC at diagnosis.

However, CS in patients with poor baseline liver function did not tend to improve over time. Similar results have been observed in previous studies on CS in HCC patients [23, 24]. Those studies found that baseline liver function has a constant effect on CS over time. Additionally, a previous study on the subclassification of BCLC stage found that patients with poor liver function had worse survival outcomes than patients with preserved liver function [39]. In the present study, the proportion of Child-Pugh class B patients in long-term (> 2 years) survivors was only 3% (3/34), and 72.7% (16/22) of Child-Pugh class B patients died within 1 year after treatment. The OS and CS of Child-Pugh class A patients was significantly higher than class B patients and standardized differences did not decrease over time between Child-Pugh class A and B patients. Therefore, we conclude that underlying liver function has a constant effect on CS over time and we should emphasize that sufficient preserved liver function at treatment initiation is an important factor for long-term prognosis.

Our study had several strengths. First, to the best of our knowledge, this study is the first to estimate CS in advanced HCC patients treated with HAIC with/without CRT, which is a non-curative loco-regional therapy. Compared to previous studies, patients in this study group had more advanced-stage HCC and worse underlying liver function [23, 24]. However, the CS estimate increased over time in both BCLC stage B and C patients with notable treatment outcomes. Therefore, clinicians can provide a hopeful message that prognosis can be improved through loco-regional therapy, even in patients with advanced-stage HCC at the time of diagnosis. Second, the new HCC staging system, the HKLC staging system, was validated. The HKLC staging system was effective at predicting patient survival. The HKLC staging system, based on data from HBV-related HCC patients with relatively preserved basal liver function, subdivides the BCLC stage and emphasizes more aggressive treatment than the BCLC staging system. The OS of patients differed significantly according to subdivided HKLC stage II, III, and IV in this population. Furthermore, the tendency of CS to increase over time was also observed in HKLC stage 2 and 3 patients. The 2-year CS of 0, 1, and 2 in HKLC stage II patients was 56.9%, 68.3%, and 82.3%, respectively, and 32.4%, 54.2%, and 75.6%, respectively, in HKLC stage III patients. The tendency of CS to increase over time has been confirmed in different staging systems, suggesting that CS estimates can be applied regardless of the staging system.

This study had some limitations. First, it had a relatively small sample size and retrospective design. The study population did not represent all patients with advanced HCC. Second, the follow-up duration was not long enough to confirm the long-term data of CS tendency. Sufficient follow-up duration and a larger sample size providing sufficient long-term survival data would lead to more accurate CS estimates. Third, this study did not analyze changes in patient quality of life and how these affected survival. Although no life-threatening serious adverse effects after HAIC with CRT were observed in this study population, medical records regarding changes in performance status (e.g., ECOG status) or quality of life (e.g., FACT-hep) were insufficient. Finally, due to the aggressiveness of the advanced stage HCC, the number of survivors declined sharply over time, so subjects to be analyzed after years were rapidly decreased. It limits clinical implication of this study. Further investigations and a well-organized prospective study are needed to overcome these limitations.

In conclusion, CS tends to increase over time in patients with BCLC stage B/C HCC treated with HAIC with or without CRT. CS may provide a more accurate prognosis and a more hopeful message to patients who have already survived with treatment.

## MATERIALS AND METHODS

### Study population

The diagnosis of HCC was based on pathological confirmation or the typical appearance of HCC, either by two dynamic imaging examinations (computed tomography [CT] and magnetic resonance imaging [MRI]) or by one dynamic technique combined with elevated serum alpha-fetoprotein (AFP) level (> 200 ng/mL).

The patients were identified using the Severance Hospital Hepatocellular Carcinoma Cohort Registry, which is an internal Web-based electronic medical record that encompasses HCC patients who were treated with anticancer therapy at the Severance Hospital, Yonsei University College of Medicine, from 2011 to 2015. A total of 228 patients were found to have received HAIC with or without CRT to treat advanced HCC. The inclusion criteria were as follows: (1) 18 years of age or older; (2) at least one measurable or evaluable HCC lesion; (3) Eastern Cooperative Oncology Group (ECOG) performance status ≤ 2; (4) no prior antitumor treatment before HAIC with or without CRT, such as TACE, surgery or radiofrequency ablation; (5) estimated life expectancy ≥ 12 weeks; (6) Child-Pugh class A or B; (7) BCLC stage B and C; and (8) other adequate organ function (absolute neutrophil count ≥ 1.5 × 10^9^/L, serum creatinine < 1.5 mg/dL or calculated creatinine clearance ≥ 60 mL/min by the Cockcroft and Gault formula, aminotransferase level less than 5 times the upper limit of normal). Finally, 181 patients were identified as eligible patients who met the enrollment criteria.

This study was approved by the local Institutional Review Board and was conducted in accordance with the principles set forth in the Declaration of Helsinki. Written informed consent was obtained from all patients

### Implantation of the port system

The femoral artery was accessed by experienced interventional radiologists using the Seldinger method. Arteriography of the celiac trunk and superior mesenteric artery was performed to evaluate hepatic arterial vascularization and portal vein patency, respectively. After detection of the HCC and its feeding artery, the tip of the catheter (Port-A-Cath1; Deltac, St. Paul, MN, USA) was placed at the common hepatic artery or proper hepatic artery under fluoroscopic guidance. The proximal end of the catheter was connected to the injection port, which was implanted into a subcutaneous pocket in the right iliac fossa. To prevent thrombosis of the catheter lumen, 10,000 units of heparin were infused via the port after each cycle of chemotherapy.

### Treatment protocols and response evaluation

Patients treated with HAIC and CRT received a total radiation dose of 45 Gy in 25 fractions over a period of 5 weeks with a concurrent hepatic arterial infusion of 5-fluorouracil (5-FU; 500 mg/day for 5 h on 5 consecutive days through an implanted port system) during the first and fifth weeks of radiotherapy. For patients treated with HAIC without CRT, 5-FU (500 mg/m^2^ for 5 h on days 1–3) and cisplatin (60 mg/m^2^ for 2 h on day 2) were administered every 4 weeks based on a previously described method [15]. In every CRT patient, CT-based three-dimensional (3D) treatment planning was performed to determine the target volumes, radiation ports, and dose prescription. The gross tumor volumes (GTVs) were defined as the tumor areas noted on the CT, including portal vein thrombosis. A minimum of 5 mm around the GTV was included in the clinical target volume. The planning target volume was designed so that the margins were individualized by observing liver position as well as liver movement at the time of simulation. The distance of diaphragmatic excursion by respiration, which was observed fluoroscopically, was added to determine the cranial–caudal margins. Standard localized HAIC and CRT protocols were maintained throughout the study period.

After 5 weeks of HAIC with CRT or 2 cycles of HAIC without CRT, treatment response was evaluated by CT or MRI according to RECIST criteria [40] and consecutive treatment such as HAIC or systemic chemotherapy was performed according to the patient’s treatment response. Objective response was defined as complete response (CR) and PR. Disease control was defined as CR, PR, and SD that maintained 16 or more weeks.

### Definition of conditional survival

Conditional survival (CS) is derived from the biostatistics concept of conditional probability [41] and can be calculated from traditional Kaplan-Meier or actuarial life table survival data. The mathematical definition of CS is expressed as follows: CS (y│x) is the probability of surviving for an additional y years, given that the patient has already survived x years. Let S(t) be the traditional actuarial survival at time t; CS can be calculated as:

$$CS\left(y|x\right)=\frac{S\left(s+y\right)}{S\left(x\right)}$$ (1)

For example, to estimate the 3-year CS for a patient who has already survived 2 years, we simply divide the survival estimate at t = 3 + 2, S(5) by the survival at t = 2, S(2).

### Statistical analysis

All statistical analyses were performed using SPSS for windows version 23.0 (SPSS Inc., Chicago, IL, USA). Student’s *t*-test was used to compare continuous variables, and the chi-square or Fisher’s exact test was used for categorical variables. Overall survival (OS) was computed from the day treatment began until the most recent follow-up or death. Survival time and rate were estimated by the Kaplan-Meier method, and differences between groups were assessed using the log-rank test. A *P* value < 0.05 was considered statistically significant.

The CS differences between groups were compared by calculating standardized differences (*d*), which were used in terms of effect size. Standardized differences were calculated as follows:

$$d=\frac{\left(Pp-Pe\right)}{\sqrt{\left[Pp\left(1-Pp\right)+Pe\left(1-Pe\right)\right]}/2}$$ (2)

where P_p_ and P_e_ denote the proportion of a binary baseline variable in two groups [42]. *d* values less than 0.1 indicate very small differences between groups; *d* values between 0.1 and 0.3 indicate small differences; *d* values between 0.3 and 0.5 indicate moderate differences; and *d* values greater than 0.5 indicate considerable differences.

## Abbreviations

BCLC: Barcelona Clinic Liver Cancer
CCRT: Concurrent chemoradiation therapy
CRT: Concurrent radiotherapy
CS: Conditional survival
CR: Complete response
HAIC: Hepatic arterial infusion chemotherapy
HBV: Hepatitis B virus
HCC: Hepatocellular carcinoma
HKLC: Hong Kong Liver Cancer
HV: Hepatic vein
OS: Overall survival
PR: Partial response
PV: Portal vein
PVT: Portal vein thrombosis
RFA: Radiofrequency ablation
SD: stable disease
TACE: Transhepatic arterial chemoembolization
